# Risk factors for postoperative recurrence of chronic subdural hematoma and construction and validation of an online dynamic nomogram prediction model

**DOI:** 10.3389/fneur.2026.1877761

**Published:** 2026-10-07

**Authors:** Xiangyu Zhang, Zhicheng Huang, Sai Li, Binghui Wu, Mingjun Li, Hongliang Wang, Bing Zhao, Zhifei Guo

**Affiliations:** Department of Neurosurgery, The Second Affiliated Hospital of Anhui Medical University, Hefei, China

**Keywords:** chronic subdural hematoma, online dynamic nomogram, postoperative recurrence, prediction model, risk factors

## Abstract

**Objective:**

To investigate factors associated with postoperative recurrence in patients with chronic subdural hematoma (CSDH) and to construct and internally validate an online dynamic nomogram for individualized recurrence-risk estimation.

**Methods:**

This retrospective single-center study included 284 surgically treated patients with CSDH between January 2018 and May 2025. Before model development, the cohort was divided at a 7:3 ratio into a training cohort (*n* = 198) and a validation cohort (*n* = 86). Univariate analysis, multivariable logistic regression, predictor selection, and nomogram construction were performed exclusively in the training cohort, whereas the validation cohort was reserved for model performance assessment. Recurrence was defined as reaccumulation or enlargement of the hematoma on postoperative CT within 6 months after surgery, accompanied by recurrent or worsening neurological symptoms, regardless of whether repeat surgery was subsequently performed.

**Results:**

Overall, 54 of 284 patients (19.01%) experienced recurrence; 33 recurrence events occurred in the training cohort and 21 in the validation cohort. Smoking history, laterality, hematoma density, greatest hematoma thickness, cerebral midline shift ≥10 mm, and postoperative pneumocephalus were statistically significant in the multivariable analysis. The AUC was 0.89 (95% CI, 0.82–0.95, sensitivity 0.82, specificity 0.85) in the training cohort and 0.74 (95% CI, 0.59–0.88, sensitivity 0.52, specificity 0.85) in the validation cohort. Bootstrap optimism correction yielded an apparent AUC of 0.892, a mean optimism of 0.021, and an optimism-corrected AUC of 0.871. In the training cohort, the Hosmer-Lemeshow test showed no statistically significant evidence of lack of fit (*χ*^2^ = 9.96, df = 8, *p* = 0.268).

**Conclusion:**

The online nomogram provides a convenient framework for individualized recurrence risk estimation. Further external multicenter validation is warranted to assess its generalizability and clinical applicability.

## Introduction

Chronic subdural hematoma (CSDH) is a collection of blood and blood-breakdown products in the subdural space, most commonly affecting older adults and often occurring after minor head trauma. CT is the principal imaging modality, while MRI can provide additional characterization in selected cases (1, 2). CSDH may cause progressive neurological deterioration through mass effect, ventricular compression, and midline shift; in severe cases, cerebral herniation may occur. Postoperative recurrence remains an important clinical problem, and clinically relevant recurrence is commonly assessed during the first several postoperative months (3). The mechanisms of recurrence are multifactorial and include persistent vascularized hematoma membranes, ongoing inflammatory and fibrinolytic activity, impaired brain re-expansion and residual subdural space, postoperative pneumocephalus, and drainage-related factors (4–7). Imaging features such as hematoma thickness, density pattern, and midline shift have also been associated with recurrence risk (8–10). Although several studies have proposed recurrence-risk models, no single model has achieved broad external validation or broader clinical application (11, 12). Therefore, we retrospectively evaluated clinical, radiological, and perioperative factors associated with postoperative CSDH recurrence and developed an online dynamic nomogram intended to provide individualized risk estimation.

## Methods

### Study population and data source

A retrospective analysis of surgically treated CSDH cases at our hospital from January 2018 to May 2025 was performed. The inclusion criteria were: (1) CSDH confirmed by CT or MRI; (2) surgical treatment for CSDH; and (3) availability of the clinical, imaging, and laboratory information required for the planned analyses. The exclusion criteria were: (1) severe systemic disease preventing surgery or follow-up; (2) missing key clinical or imaging variables required for analysis; and (3) acute or subacute subdural hematoma or non-surgical management. Patients with important missing predictor or outcome data were excluded before the final study cohort was established; no imputation was performed within the 284 included cases. A total of 284 patients formed the final cohort. All 284 included patients completed the 6-month postoperative follow-up required for outcome ascertainment.

### Variables and outcome assessment

Variables extracted from the medical records included age, sex, diabetes mellitus, smoking history, drinking history, hypertension, aspirin-use history, platelet count <100 × 10^9/L, hematoma laterality, hematoma density, greatest hematoma thickness, cerebral midline shift ≥10 mm, postoperative pneumocephalus, preoperative liver dysfunction, preoperative hypoalbuminemia, drainage-tube depth, neuroendoscopic assistance, an MRI gradient sign, PT-INR, burr-hole diameter >1 cm, fibrinogen (FIB), thrombin time (TT), and APTT ≥40 s. Laterality was categorized as unilateral or bilateral; hematoma density as low or mixed density; greatest hematoma thickness (mm) and drainage-tube depth (mm) were treated as continuous variables. Cerebral midline shift was dichotomized as No (<10 mm) and Yes (≥10 mm). Postoperative pneumocephalus was recorded as a binary variable (No/Yes). The MRI gradient sign was defined as the presence or absence of an intrahematomal signal gradient recorded on preoperative MRI. Burr-hole diameter >1 cm was recorded as a binary variable (No/Yes).

Primary outcome definition: Recurrence was defined as reaccumulation or enlargement of the hematoma on postoperative CT within 6 months after surgery, accompanied by recurrent or worsening neurological symptoms, regardless of whether repeat surgery was subsequently performed.

### Surgical procedures and postoperative assessment

All patients underwent burr-hole drainage under local or general anesthesia. For unilateral chronic subdural hematoma, a single burr hole was routinely created at the site of maximal hematoma thickness identified on preoperative CT. For other cases, the number and location of burr holes were determined by the attending neurosurgeon according to hematoma extent and distribution. Intraoperative irrigation with warmed normal saline was performed until the effluent was clear. A closed-system subdural drainage catheter (14-Fr or 16-Fr silicone catheter) was placed in the subdural space and connected to a sterile closed drainage bag. The drainage tube was advanced approximately 2–5 cm into the hematoma cavity, with the tip positioned anteriorly or posteriorly according to the surgeon’s preference. Postoperative drainage was maintained for 24–72 h, with the drainage bag positioned approximately 10–20 cm below the level of the external auditory meatus to facilitate gravity-dependent drainage. No membranectomy or inner membrane fenestration was performed in any patient. All patients underwent routine postoperative CT within 24–48 h to assess hematoma evacuation, brain re-expansion, and postoperative intracranial air. Pneumocephalus was defined as any visible intracranial gas on postoperative CT and was analyzed as a binary variable (No/Yes). In our clinical practice, most cases of postoperative pneumocephalus were managed conservatively and allowed to resolve spontaneously with routine postoperative care. No patient required surgical intervention specifically for pneumocephalus.

### Statistical methods

All statistical analyses were performed using R version 4.4.0. Continuous variables are presented as mean ± standard deviation and categorical variables as number (percentage); odds ratios (ORs) are reported with 95% confidence intervals (CIs). Before model development, the complete cohort of 284 patients was divided at a 7:3 ratio into a training cohort (*n* = 198) and a validation cohort (*n* = 86). Univariate analyses were performed exclusively in the training cohort, and variables with *p* < 0.05 were entered into the multivariable logistic regression model. The validation cohort was not used for univariate screening, coefficient estimation, predictor selection, or model fitting and was reserved for evaluation of model performance. Six predictors remained statistically significant in the multivariable logistic regression model; the coefficient estimates for these six predictors from that multivariable model were used to construct the static and online dynamic nomograms. To evaluate the robustness of coefficient estimates under the condition of a limited number of recurrence events, a sensitivity analysis was performed using Firth penalized logistic regression. The same six predictors included in the primary nomogram were assessed using penalized likelihood estimation. Variance inflation factors (VIFs) were calculated among the six predictors incorporated into the nomogram to assess multicollinearity. Model discrimination was evaluated using ROC curves and AUCs with 95% CIs, together with sensitivity and specificity. Calibration was assessed using calibration curves with 1,000 bootstrap resamples and the Hosmer-Lemeshow goodness-of-fit test. Bootstrap internal validation with 1,000 resamples was also used to estimate mean optimism in AUC and to obtain an optimism-corrected AUC. Decision curve analysis (DCA) and a clinical impact curve (CIC) were used as complementary graphical assessments of potential clinical utility. Because 10 predictors met the univariate *p* < 0.05 criterion and were entered into the multivariable model, the 33 recurrence events in the training cohort corresponded to approximately 3.3 recurrence events per predictor entered into the multivariable model. The model was developed using routinely available clinical and imaging variables, and further external validation is required to evaluate its generalizability. The dynamic nomogram was implemented in R using the Shiny framework and deployed through shinyapps.io at https://wdbldwhzc.shinyapps.io/DynNomapp/. *p* < 0.05 was considered statistically significant.

## Results

A total of 284 patients met the inclusion criteria; 54 patients (19.01%) experienced postoperative recurrence. Before model development, the cohort was divided into a training cohort of 198 patients (165 non-recurrences and 33 recurrences) and a validation cohort of 86 patients (65 non-recurrences and 21 recurrences). All analyses reported in Tables 1–3, including univariate screening and multivariable regression, were performed in the training cohort; the validation cohort was reserved solely for model performance assessment. Thus, the smaller sample size in Tables 1–3 reflects the planned training/validation split rather than additional exclusion for missing data.

**Table 1:** Baseline characteristics and univariate analysis of postoperative recurrence in the training cohort.

Variables	Training cohort (*n* = 198)	Non-recurrence (*n* = 165)	Recurrence (*n* = 33)	Statistic	*p*
Age, Mean ± SD	67.56 ± 11.69	67.76 ± 11.85	66.52 ± 11.01	*t* = 0.56	0.577
Greatest thickness (mm), Mean ± SD	19.18 ± 6.50	18.26 ± 5.71	23.75 ± 8.22	*t* = −3.66	**<0.001**
PT INR, Mean ± SD	1.04 ± 0.13	1.05 ± 0.14	1.01 ± 0.09	*t* = 2.42	**0.018**
TT, Mean ± SD	17.53 ± 2.55	17.70 ± 1.98	16.69 ± 4.35	*t* = 1.31	0.200
FIB, Mean ± SD	2.89 ± 0.68	2.87 ± 0.70	2.97 ± 0.60	*t* = −0.78	0.435
Drainage tube depth (mm), Mean ± SD	27.34 ± 9.15	26.49 ± 8.24	31.58 ± 12.03	*t* = −2.32	**0.026**
Sex, *n* (%)				*χ*^2^ = 0.00	1.000
F	78 (39.39)	65 (39.39)	13 (39.39)		
M	120 (60.61)	100 (60.61)	20 (60.61)		
Drinking history, *n* (%)				*χ*^2^ = 0.00	0.949
No	95 (47.98)	79 (47.88)	16 (48.48)		
Yes	103 (52.02)	86 (52.12)	17 (51.52)		
Smoking history, *n* (%)				*χ*^2^ = 13.04	**<0.001**
No	146 (73.74)	130 (78.79)	16 (48.48)		
Yes	52 (26.26)	35 (21.21)	17 (51.52)		
Diabetes mellitus, *n* (%)				*χ*^2^ = 0.00	1.000
No	189 (95.45)	158 (95.76)	31 (93.94)		
Yes	9 (4.55)	7 (4.24)	2 (6.06)		
Hypertension, *n* (%)				*χ*^2^ = 1.47	0.225
No	132 (66.67)	113 (68.48)	19 (57.58)		
Yes	66 (33.33)	52 (31.52)	14 (42.42)		
History of aspirin use, *n* (%)				*χ*^2^ = 6.33	**0.012**
No	169 (85.35)	146 (88.48)	23 (69.70)		
Yes	29 (14.65)	19 (11.52)	10 (30.30)		
Platelet count <100 × 10^9/L, *n* (%)				-	0.130
No	194 (97.98)	163 (98.79)	31 (93.94)		
Yes	4 (2.02)	2 (1.21)	2 (6.06)		
Laterality, *n* (%)				*χ*^2^ = 4.94	**0.026**
Bilateral	27 (13.64)	18 (10.91)	9 (27.27)		
Unilateral	171 (86.36)	147 (89.09)	24 (72.73)		
Hematoma density, *n* (%)				*χ*^2^ = 14.77	**<0.001**
Low density	157 (79.29)	139 (84.24)	18 (54.55)		
Mixed density	41 (20.71)	26 (15.76)	15 (45.45)		
Cerebral midline shift ≥10 mm, *n* (%)				*χ*^2^ = 14.85	**<0.001**
No	144 (72.73)	129 (78.18)	15 (45.45)		
Yes	54 (27.27)	36 (21.82)	18 (54.55)		
Neuroendoscopic assistance, *n* (%)				*χ*^2^ = 0.37	0.541
0	154 (77.78)	127 (76.97)	27 (81.82)		
1	44 (22.22)	38 (23.03)	6 (18.18)		
Preoperative liver dysfunction, *n* (%)				*χ*^2^ = 1.05	0.305
No	185 (93.43)	156 (94.55)	29 (87.88)		
Yes	13 (6.57)	9 (5.45)	4 (12.12)		
MRI gradient sign, *n* (%)				-	1.000
No	196 (98.99)	163 (98.79)	33 (100.00)		
Yes	2 (1.01)	2 (1.21)	0 (0.00)		
Preoperative hypoalbuminemia, *n* (%)				*χ*^2^ = 5.36	**0.021**
0	151 (76.26)	131 (79.39)	20 (60.61)		
1	47 (23.74)	34 (20.61)	13 (39.39)		
APTT ≥40 s, *n* (%)				*χ*^2^ = 0.01	0.919
0	176 (88.89)	146 (88.48)	30 (90.91)		
1	22 (11.11)	19 (11.52)	3 (9.09)		
Burr-hole diameter >1 cm, *n* (%)				-	0.195
0	193 (97.47)	162 (98.18)	31 (93.94)		
1	5 (2.53)	3 (1.82)	2 (6.06)		
Pneumocephalus, *n* (%)				*χ*^2^ = 28.64	**<0.001**
No	154 (77.78)	140 (84.85)	14 (42.42)		
Yes	44 (22.22)	25 (15.15)	19 (57.58)		

*t*, *t*-test, *χ*^2^, Chi-square test; −, Fisher exact; SD, standard deviation.

Bold values indicate statistically significant values (*p* < 0.05).

**Table 2:** Predictor coding and definitions used in the multivariable analysis.

Variable name	Code	Definition/assignment
Smoking history	X1	Yes = 1; No = 0
Laterality	X2	Unilateral = 1; Bilateral = 0
Hematoma density	X3	Mixed density = 1; Low density = 0
Greatest hematoma thickness (mm)	X4	Continuous variable (mm)
Cerebral midline shift ≥10 mm	X5	Yes = 1 (≥10 mm); No = 0 (<10 mm)
Pneumocephalus	X6	Yes = 1; No = 0
PT-INR	X7	Continuous variable
Preoperative hypoalbuminemia	X8	Yes = 1; No = 0
History of aspirin use	X9	Yes = 1; No = 0
Drainage-tube depth (mm)	X10	Continuous variable (mm)

Laterality was coded as unilateral = 1 and bilateral = 0. Cerebral midline shift was coded as Yes = 1 for ≥10 mm and No = 0 for <10 mm. Greatest hematoma thickness (mm), PT-INR, and drainage-tube depth (mm) were analyzed as continuous variables; the remaining listed predictors were categorical variables coded as shown.

**Table 3:** Multivariable logistic regression results in the training cohort.

Variables	*β*	S.E	*Z*	*p*	OR (95% CI)
Intercept	−2.17	2.29	−0.95	0.344	0.11 (<0.01–10.22)
Greatest hematoma thickness	0.08	0.04	2.17	**0.030**	1.09 (1.01–1.17)
PT-INR	−2.07	2.07	−1.00	0.318	0.13 (<0.01–7.29)
Drainage-tube depth	0.00	0.02	0.03	0.978	1.00 (0.95–1.05)
Smoking history					
No					1.00 (Reference)
Yes	1.03	0.53	1.96	**0.049**	2.81 (1.01–7.87)
History of aspirin use					
No					1.00 (Reference)
Yes	1.11	0.58	1.90	0.058	3.03 (0.96–9.52)
Laterality					
Bilateral					1.00 (Reference)
Unilateral	−1.49	0.63	−2.35	**0.019**	0.22 (0.06–0.78)
Hematoma density					
Low density					1.00 (Reference)
Mixed density	1.38	0.53	2.58	**0.010**	3.96 (1.39–11.28)
Cerebral midline shift ≥10 mm					
No					1.00 (Reference)
Yes	1.23	0.53	2.30	**0.022**	3.41 (1.20–9.73)
Preoperative hypoalbuminemia	1.02	0.55	1.85	0.065	2.78 (0.94–8.21)
Pneumocephalus					
No					1.00 (Reference)
Yes	1.10	0.53	2.07	**0.038**	3.00 (1.06–8.49)

Confidence-interval lower bounds below 0.01 are reported as <0.01 rather than 0.00 because odds ratios are strictly positive and their confidence intervals cannot include zero. The six statistically significant predictors incorporated into the nomogram were smoking history, laterality, hematoma density, greatest hematoma thickness, cerebral midline shift ≥10 mm, and postoperative pneumocephalus. The wide confidence intervals for some estimates reflect substantial imprecision and should be interpreted cautiously. OR, odds ratio; CI, confidence interval.

Bold values indicate statistically significant values (*p* < 0.05).

### Univariate analysis of recurrence-related factors in the training cohort

In the univariate analysis, several clinical, laboratory, radiological, and postoperative factors were significantly associated with postoperative recurrence in the training cohort (Table 1). Variables with *p-*values <0.05 included PT-INR, smoking history, aspirin use, laterality, hematoma density, greatest hematoma thickness, cerebral midline shift ≥10 mm, preoperative hypoalbuminemia, drainage-tube depth, and postoperative pneumocephalus. These variables were subsequently considered for inclusion in the multivariable logistic regression analysis.

### Multivariable analysis of postoperative recurrence in the training cohort

Postoperative recurrence was the dependent variable. Ten variables with *p* < 0.05 in the univariate analysis were entered into multivariable logistic regression within the training cohort. Smoking history, laterality, hematoma density, greatest hematoma thickness, cerebral midline shift ≥10 mm, and postoperative pneumocephalus were statistically significant in the multivariable analysis (Table 3). The coefficient estimates for these six statistically significant predictors from the multivariable model were used to construct the nomogram (see Figures 1, 2).

**Figure 1:**
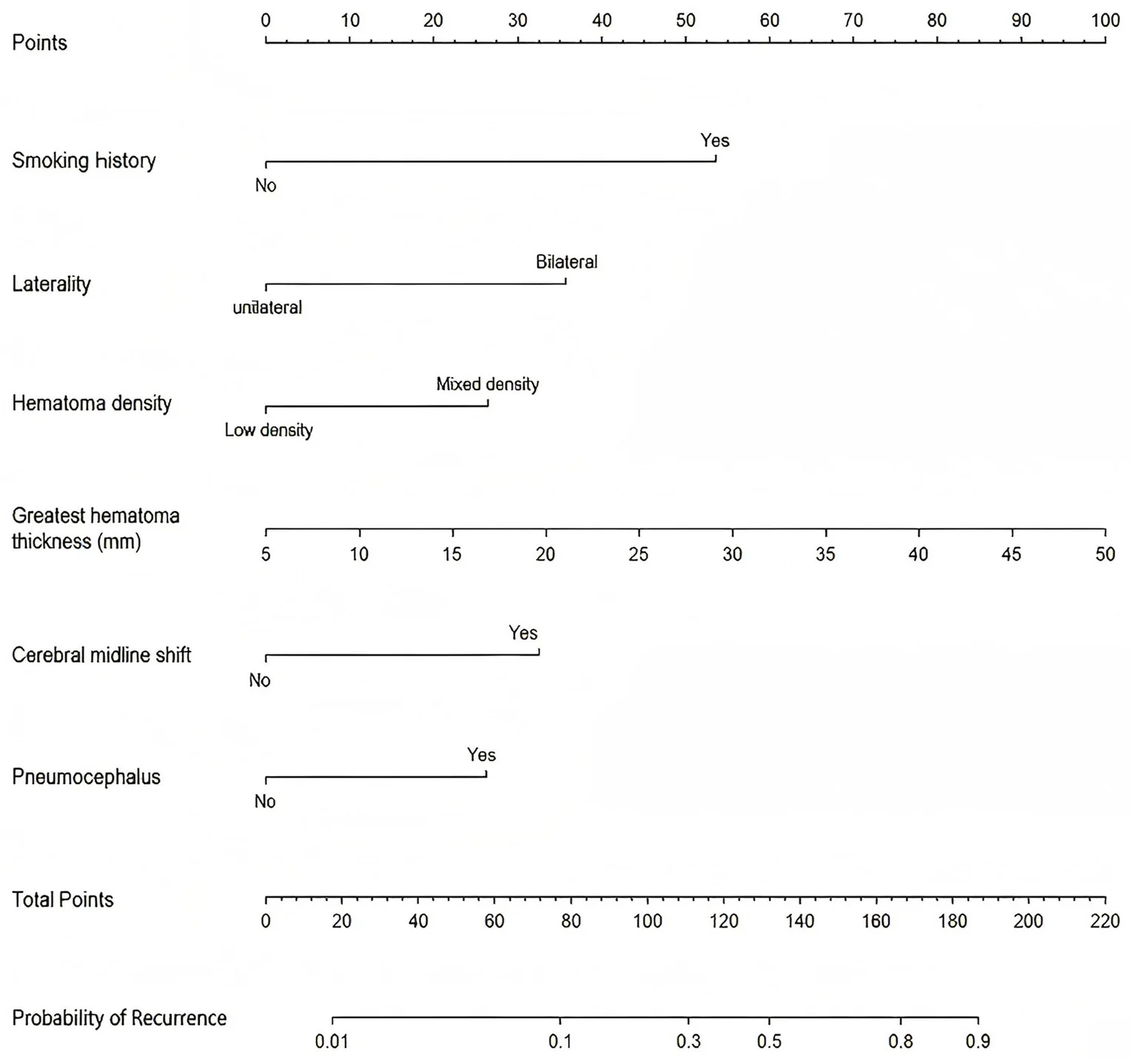
Nomogram for estimating postoperative recurrence of CSDH. The nomogram incorporates six predictors: smoking history, hematoma laterality, hematoma density, greatest hematoma thickness (mm), cerebral midline shift, and postoperative pneumocephalus. Cerebral midline shift was dichotomized at 10 mm as specified in the Methods.

**Figure 2:**
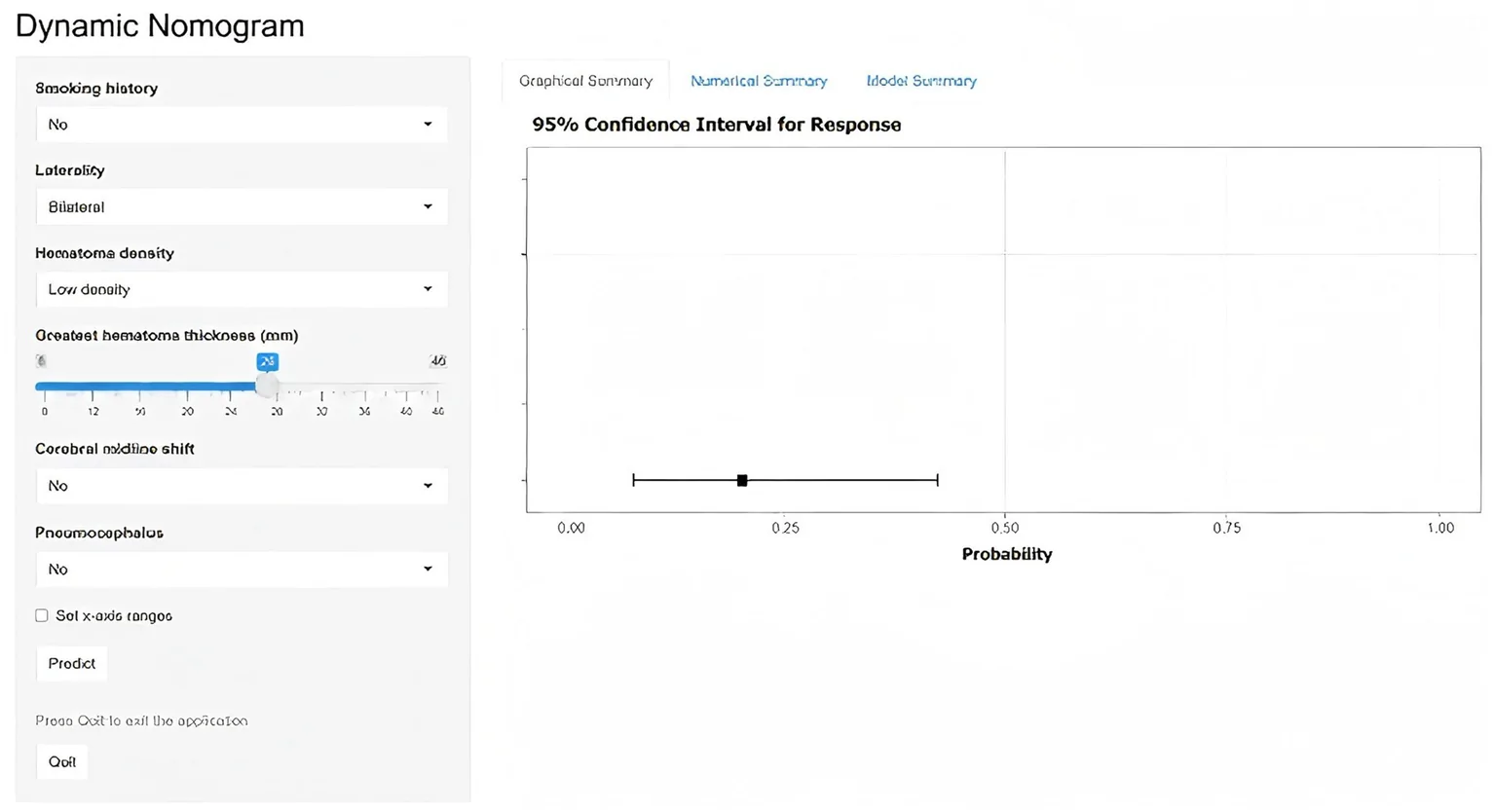
Online dynamic nomogram for individualized postoperative recurrence-risk estimation. The web application uses the same six predictors as the static nomogram and is publicly accessible at https://wdbldwhzc.shinyapps.io/DynNomapp/.

### Construction and validation of a nomogram prediction model for predicting postoperative recurrence of chronic subdural hematoma

The nomogram was developed using the coefficient estimates for the six statistically significant predictors from the training-cohort multivariable model and was evaluated in the validation cohort. In the training cohort, the AUC was 0.89 (95% CI, 0.82–0.95), sensitivity was 0.82, and specificity was 0.85 (Figure 3). In the validation cohort, the AUC decreased to 0.74 (95% CI, 0.59–0.88), sensitivity to 0.52, while specificity remained 0.85 (Figure 4). Thus, discrimination was good in the training cohort but moderate in the validation cohort, with a notable reduction in sensitivity. Bootstrap internal validation with 1,000 resamples yielded an apparent AUC of 0.892, a mean estimated optimism of 0.021, and an optimism-corrected AUC of 0.871. The relatively small reduction after optimism correction suggests limited optimism in the apparent discriminatory performance, although overfitting cannot be excluded because of the limited number of recurrence events. In the training cohort, the Hosmer-Lemeshow goodness-of-fit test showed no statistically significant evidence of lack of fit (*χ*^2^ = 9.96, df = 8, *p* = 0.268). Calibration curves showed reasonable agreement between predicted and observed probabilities in the training cohort, with greater deviation in the validation cohort (Figures 5, 6). DCA showed positive net benefit relative to the treat-all and treat-none strategies across observed threshold probability ranges of approximately 0.04–0.93 in the training cohort, with the benefit more evident from approximately 0.04–0.54, and approximately 0.13–0.69 in the validation cohort, with the benefit more evident from approximately 0.13–0.61 (Figures 7, 8). These ranges are descriptive results from the DCA and were not prespecified or externally validated as clinical decision cutoffs. The CIC (Figure 9) is presented for the training cohort and shows, per 1,000 patients, the number classified as high risk and the corresponding number of observed recurrence events among those classified as high risk across threshold probabilities. Sensitivity analyses were performed to assess the robustness of the identified predictors. An exploratory parsimonious sensitivity analysis using a reduced three-predictor model showed comparable predictive performance with an increased EPV, providing additional information regarding model robustness (Supplementary Tables S1–S3). In the Firth penalized logistic regression analysis using the same six predictors included in the primary nomogram, all predictors retained consistent directions of association and remained statistically significant (Supplementary Table S4). VIF analysis showed values ranging from 1.037 to 1.150 among the evaluated predictors, suggesting that substantial multicollinearity was unlikely to have influenced the regression estimates. Taken together, these findings support the potential clinical utility of the nomogram and highlight the importance of further external validation.

**Figure 3:**
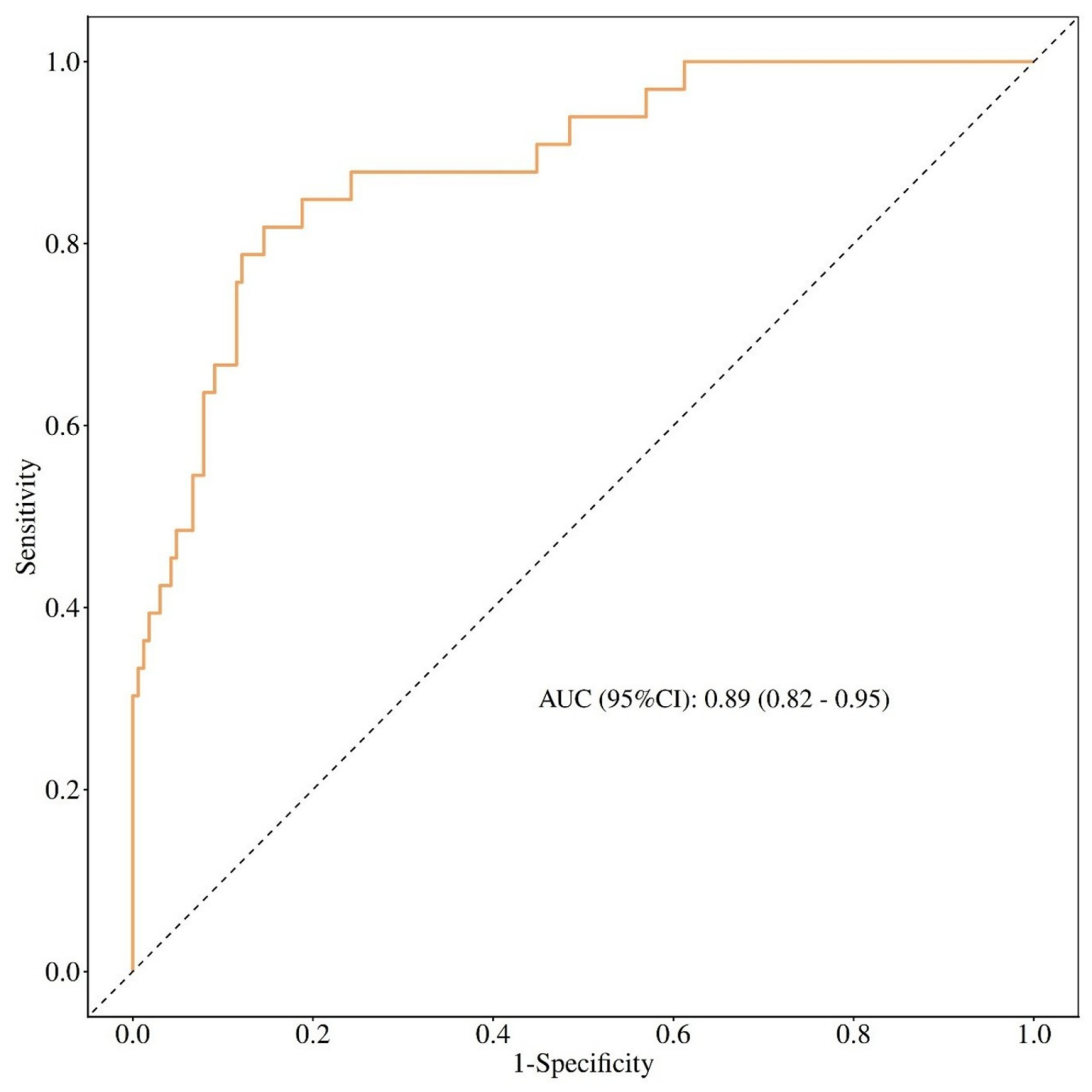
Receiver operating characteristic (ROC) curve of the prediction model in the training cohort (AUC 0.89, 95% CI 0.82–0.95).

**Figure 4:**
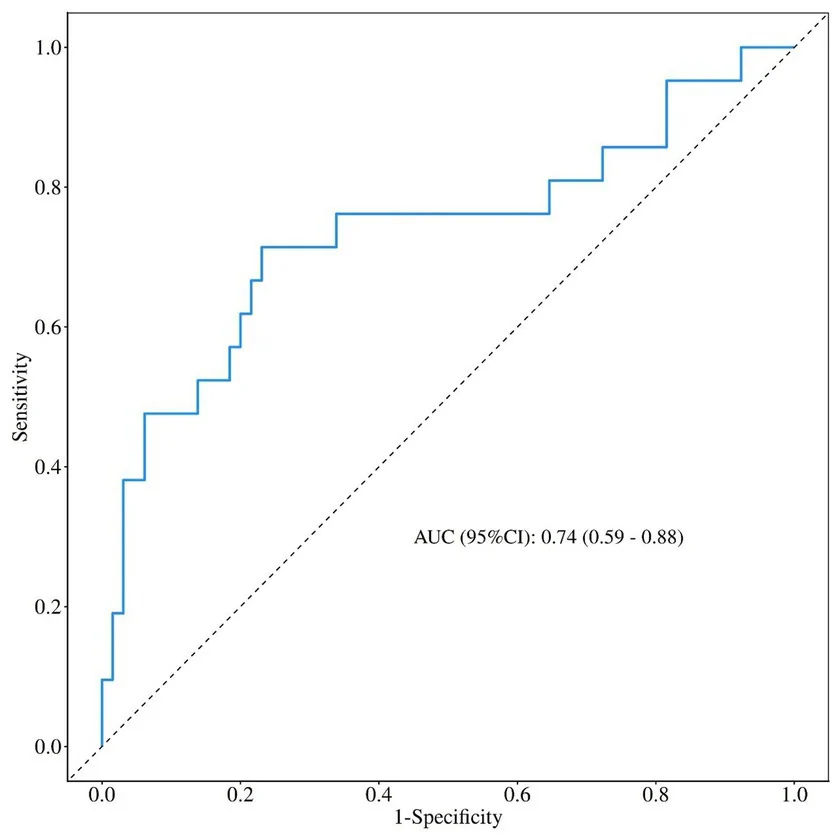
Receiver operating characteristic (ROC) curve of the prediction model in the validation cohort (AUC 0.74, 95% CI 0.59–0.88).

**Figure 5:**
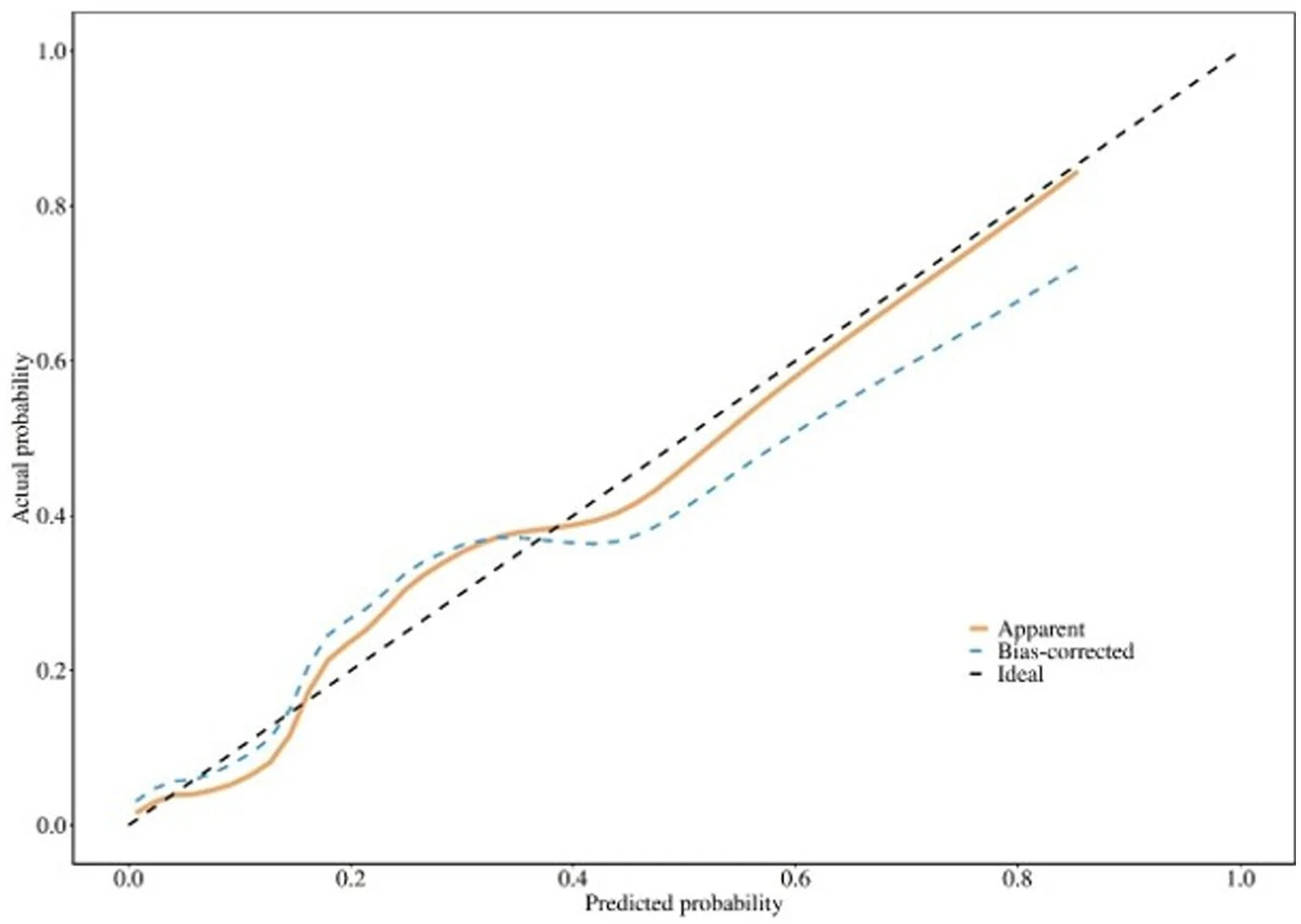
Calibration curve of the nomogram in the training cohort. The diagonal line represents perfect calibration; the apparent and bootstrap-corrected curves show observed model calibration.

**Figure 6:**
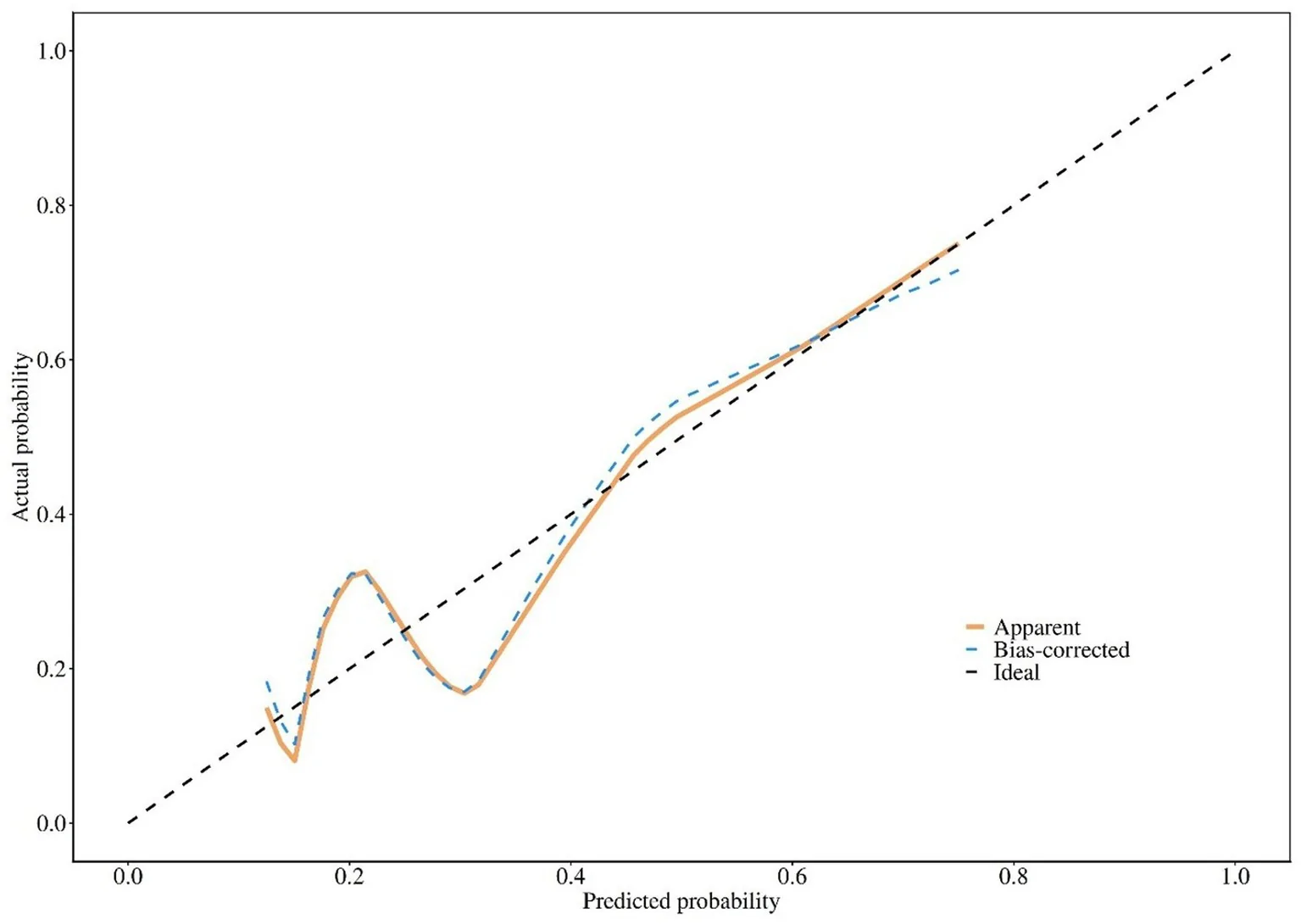
Calibration curve of the nomogram in the validation cohort. The diagonal line represents perfect calibration; the apparent and bias-corrected curves show agreement between predicted and observed recurrence probabilities.

**Figure 7:**
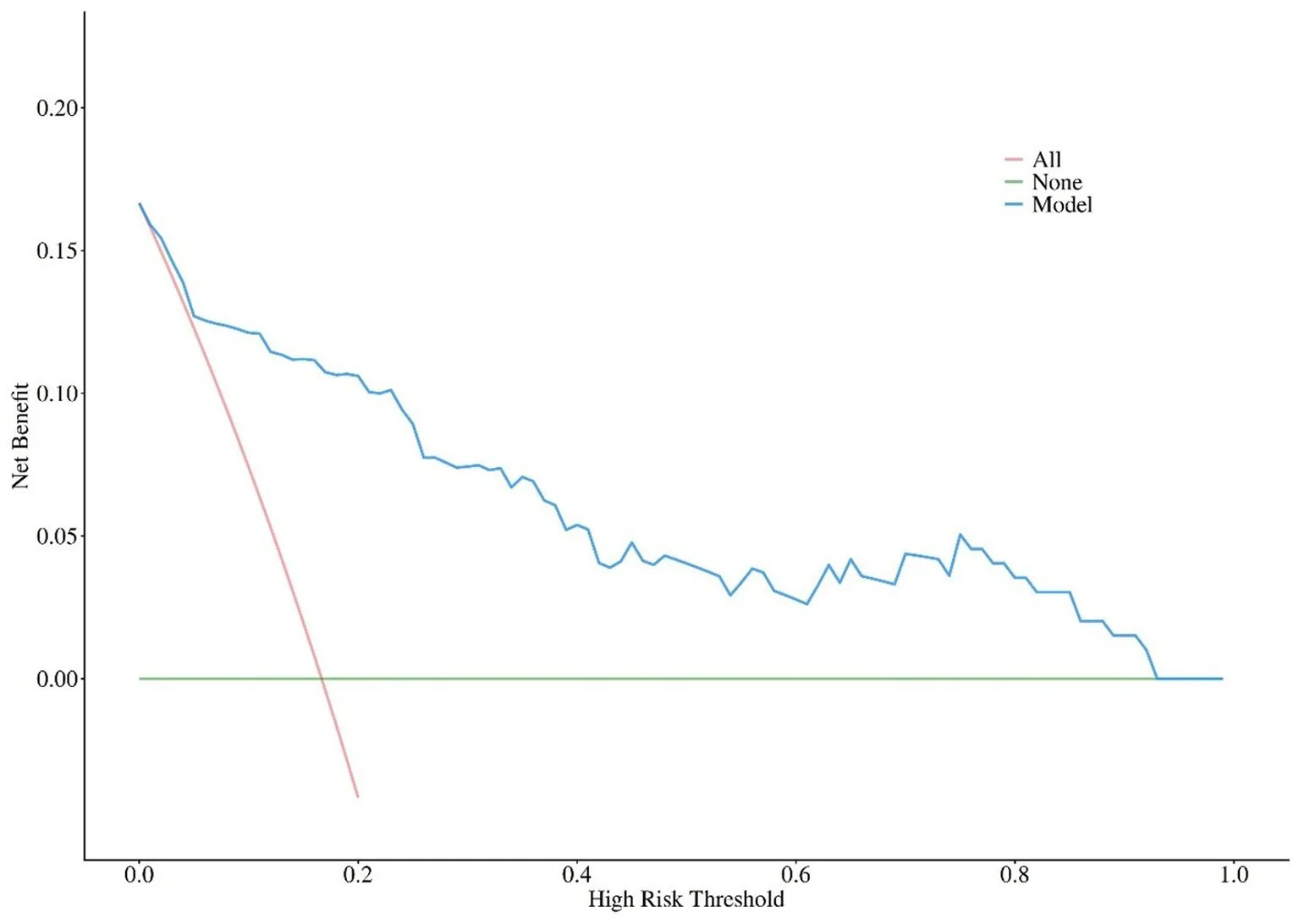
Decision curve analysis of the prediction model in the training cohort. Relative to the treat-all and treat-none strategies, the model showed positive net benefit across an observed threshold probability range of approximately 0.04–0.93, with the benefit more evident from approximately 0.04–0.54.

**Figure 8:**
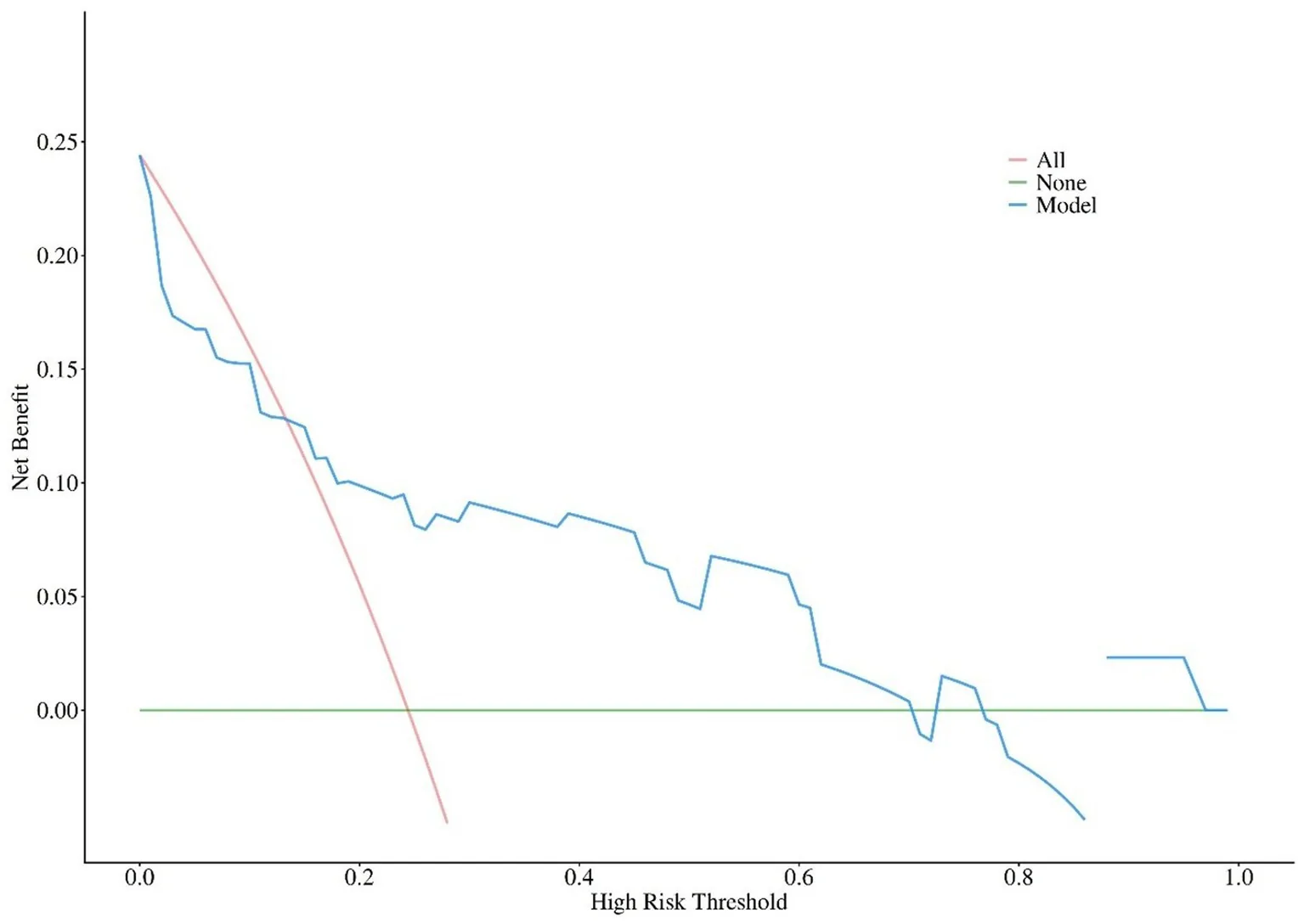
Decision curve analysis of the prediction model in the validation cohort. Relative to the treat-all and treat-none strategies, the model showed positive net benefit across an observed threshold probability range of approximately 0.13–0.69, with the benefit more evident from approximately 0.13–0.61.

**Figure 9:**
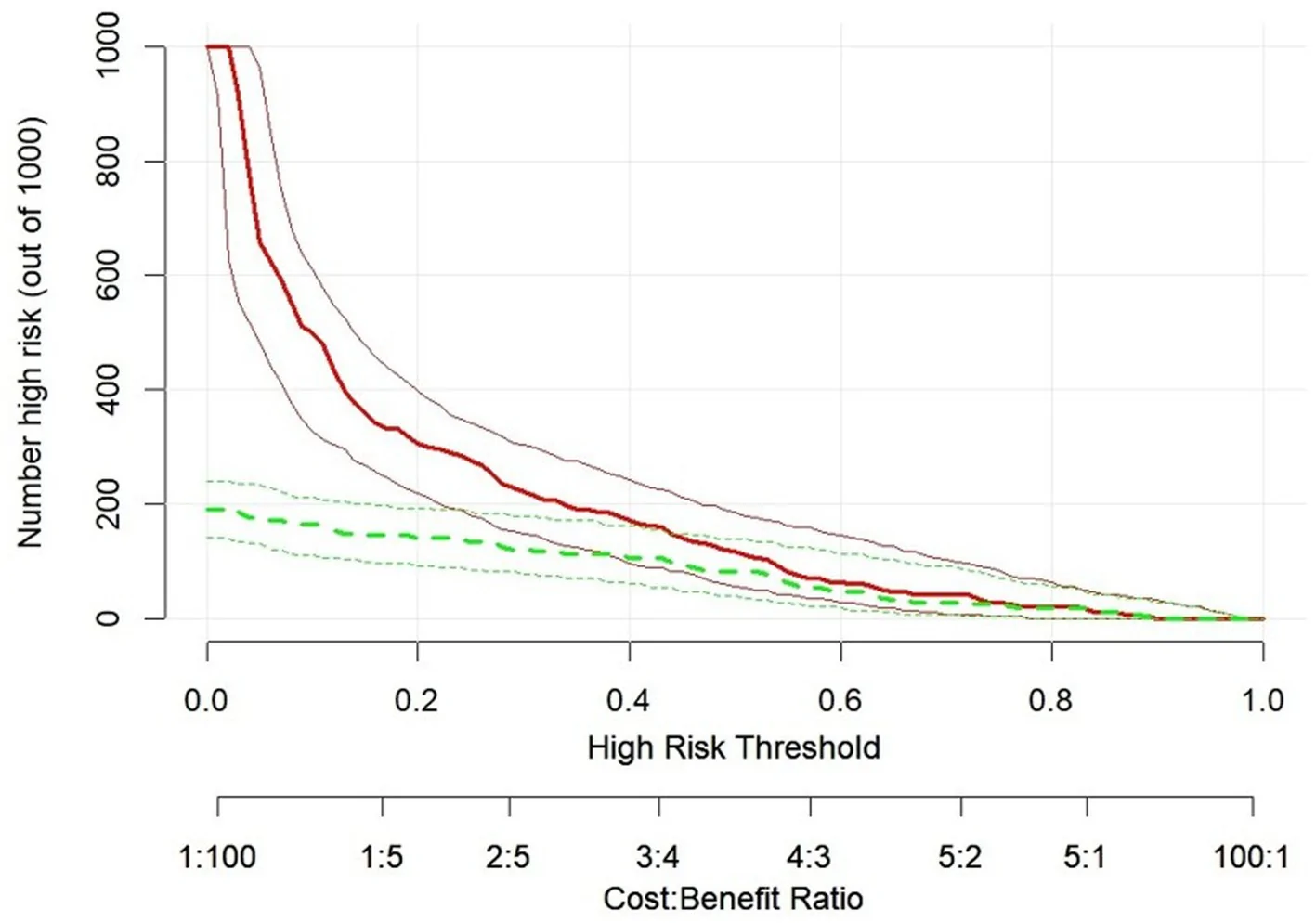
Clinical impact curve (CIC) in the training cohort. The curve displays, per 1,000 patients, the number classified as high risk and the corresponding number of observed recurrence events among those classified as high risk across threshold probabilities.

## Discussion

In this single-center retrospective study of 284 surgically treated patients with CSDH, we evaluated clinical, radiological, and perioperative factors associated with recurrence and developed a web-accessible nomogram. The primary value of this approach is the provision of an accessible individualized risk estimate based on routinely available clinical and imaging variables. The model showed good discrimination in the training cohort and maintained moderate performance in the internal validation cohort. Further external validation will be important to assess its generalizability across different clinical settings.

Smoking history remained statistically significant in our multivariable analysis (*p* = 0.049), whereas aspirin use showed only a marginal association (*p* = 0.058). A large CSDH cohort identified smoking as an independent predictor of recurrence (13), providing supportive clinical context for our finding, although the association should not be interpreted as causal. Aspirin should not be described as a confirmed independent predictor in the present cohort. Previous meta-analytic evidence has suggested that antithrombotic therapy, including antiplatelet therapy, may be associated with an increased risk of CSDH recurrence (14); however, the marginal result in our cohort warrants cautious interpretation and confirmation in larger, better-powered studies. In practical terms, smoking history may help identify a subgroup with higher observed recurrence risk in this cohort (OR = 2.81, 95% CI 1.01–7.87), but the retrospective design and limited event count preclude causal interpretation.

Radiological characteristics remain central to recurrence-risk assessment. Quantitative imaging studies and systematic reviews have associated mixed or hyperdense hematoma components, greater hematoma thickness or volume, and greater midline shift with postoperative recurrence (8–10, 15). In our cohort, mixed density, greater thickness, and cerebral midline shift ≥10 mm were independently associated with recurrence. Postoperative pneumocephalus was also significant. Recent evidence further supports the association between postoperative intracranial air and CSDH recurrence. Alenezi et al. (6) reported postoperative pneumocephalus as a predictor of recurrence, and Scala et al. (16) analyzed 460 surgically treated patients and found postoperative pneumocephalus volume to be independently associated with recurrence while comparing closed drainage with standard irrigation. Earlier observational work likewise linked postoperative subdural air with recurrence in selected patients (17). These findings are consistent with our observation and suggest that postoperative air may represent both a prognostic marker and a potentially modifiable perioperative factor. Previous studies have suggested that postoperative pneumocephalus is associated with CSDH recurrence, although the strength of this association varies across cohorts and may depend on differences in measurement methods and patient characteristics.

Bilateral CSDH was associated with a higher recurrence probability in our cohort. Systematic reviews have reported that bilateral disease may be associated with recurrence in some datasets, although the magnitude and consistency of this association vary across studies (9, 15). This finding should therefore be interpreted together with other radiological and procedural factors rather than as an isolated determinant of recurrence.

Surgical technique is another important source of heterogeneity in CSDH recurrence. Randomized evidence has shown that placement of a postoperative subdural drain after burr-hole evacuation reduces recurrence compared with no drain (7). A network meta-analysis comparing multiple surgical techniques suggested differences in recurrence and reoperation across burr-hole, twist-drill, mini-craniotomy, and craniotomy strategies (18), while cohort data also indicate that burr-hole location may influence recurrence risk (19). For selected organized or recurrent CSDH, craniotomy with membranectomy has been associated with relatively low pooled recurrence rates, although the evidence is heterogeneous and does not establish routine superiority over burr-hole drainage (20). In our cohort, all patients underwent burr-hole drainage with closed-system subdural drainage. Although the number and location of burr holes, drainage depth, and drainage duration may influence recurrence risk, these procedural factors were not completely standardized and should be considered more systematically in future prediction studies.

Comparison with previously published prediction models. Chen et al. (11) developed a six-predictor nomogram in a larger cohort of 431 patients and reported a training AUC of 0.896 with 1,000-bootstrap internal validation. Fang et al. (12) subsequently combined clinical and radiomics features with machine-learning methods and reported strong performance with external validation from an additional institution. Compared with these approaches, the potential advantage of the present model is its use of routinely available clinical and imaging variables together with a simple online interface, rather than superior discrimination. The validation performance of our model should be interpreted in the context of cohort characteristics, event frequency, and validation design. Compared with more complex approaches incorporating advanced imaging or machine-learning features, the present nomogram emphasizes accessibility by using routinely available clinical and radiological variables. The incremental clinical value of the dynamic nomogram therefore lies primarily in accessibility and ease of individualized risk display, while its transportability remains unproven.

## Limitations

This study has several limitations. First, its retrospective single-center design introduces potential selection bias, residual confounding, and center-specific practice effects. Patients with important missing clinical or imaging variables were excluded before formation of the final cohort; although no imputation was required within the included 284 cases, complete-case inclusion may reduce representativeness. Second, only 54 recurrence events occurred overall and 33 in the training cohort. Ten predictors met the univariate *p* < 0.05 criterion and were entered into the multivariable logistic regression model, corresponding to approximately 3.3 recurrence events per predictor. Although six predictors were statistically significant and incorporated into the nomogram, this post-selection subset does not remove the event-count concern arising during multivariable model development. The low event-to-predictor ratio is below traditional rule-of-thumb recommendations for logistic regression and increases the risk of overfitting and unstable coefficient estimates (21). Contemporary prediction-model guidance emphasizes that required sample size should also consider the number of candidate predictor parameters, outcome frequency, and anticipated model performance rather than relying on a fixed EPV rule alone (22). Bootstrap internal validation showed a mean optimism of 0.021, with the apparent AUC decreasing from 0.892 to an optimism-corrected value of 0.871, suggesting that the apparent discrimination was not markedly optimistic. Nevertheless, bootstrap correction cannot fully compensate for the limited development-event count, and the difference between training and validation performance highlights the importance of further evaluation in larger cohorts. Third, although all patients underwent burr-hole drainage with closed-system drainage, procedural details were not completely standardized: the number and location of burr holes, catheter position and depth, drainage duration, and selective inner-membrane fenestration varied according to clinical circumstances and surgeon preference. These differences may influence recurrence and introduce residual confounding. Fourth, the validation cohort was internal and relatively small; the difference in performance between cohorts indicates the need for further evaluation of model transportability. Fifth, postoperative pneumocephalus was analyzed only as a binary variable rather than by standardized volumetric measurement. Finally, the model has not undergone external multicenter validation. The present nomogram represents an initial individualized risk-stratification tool. External multicenter validation in larger prospective cohorts will be necessary to further evaluate its generalizability, calibration, discrimination, and clinical utility.

## Conclusion

In conclusion, we developed and internally validated a nomogram for individualized prediction of CSDH recurrence after burr-hole drainage based on routinely available clinical and imaging variables. The model demonstrated good discrimination in the training cohort and maintained acceptable performance in the internal validation cohort, supporting its potential utility for recurrence risk stratification. Further external validation in larger multicenter cohorts is warranted to evaluate its generalizability and facilitate future clinical application.

Future multicenter prospective studies with standardized surgical protocols, variable definitions, and independent external validation are warranted to further evaluate model generalizability and clinical utility. Incorporation of objective intraoperative parameters and quantitative imaging biomarkers may further improve model accuracy and clinical utility.

## Data Availability

The raw data supporting the conclusions of this article will be made available by the authors, without undue reservation.
