# Supplementary material for: Risk factors for postoperative recurrence of chronic subdural hematoma and construction and validation of an online dynamic nomogram prediction model
Source: Front Neurol. 2026 Oct 7;17:1877761. doi: 10.3389/fneur.2026.1877761 (PMC13683128; doi:10.3389/fneur.2026.1877761)
Supplement: Supplementary file 1 [file Supplementary_file_1.DOCX]

Supplementary Material

**Exploratory parsimonious sensitivity analysis in the training cohort**

# Methods

To further examine the robustness of the primary analysis under a more favorable event-to-predictor ratio, an exploratory parsimonious sensitivity analysis was performed within the original training cohort (n=198; 33 recurrence events). This analysis was designed as a secondary robustness assessment and did not replace the primary six-predictor nomogram or re-evaluate the held-out validation cohort. Following backward stepwise logistic regression using the Akaike information criterion (AIC) as the selection criterion, six candidate variables were retained and ranked by P value. The three variables with the smallest P values—smoking history, hematoma laterality, and greatest hematoma thickness—were retained in the parsimonious model. Apparent discrimination was summarized by the receiver operating characteristic (ROC) area under the curve (AUC), and decision curve analysis (DCA) was used to describe the observed threshold-probability range with positive net benefit.

# Results

The reduction-stage ranking is shown in Table S1. With 33 recurrence events and three predictors, the final parsimonious model had an events-per-variable (EPV) value of 11. The fitted three-predictor model retained smoking history, hematoma laterality, and greatest hematoma thickness. Model coefficients and corresponding odds ratios are shown in Table S2.

**Table S1. Variable ranking among retained candidates after model reduction**

| **Variable** | **Estimate** | **P value** | **Rank** | **Selected** |
| --- | --- | --- | --- | --- |
| Smoking history | 2.5535 | 0.000010 | 1 | Yes |
| Laterality (unilateral) | -2.3608 | 0.000353 | 2 | Yes |
| Greatest hematoma thickness | 0.0955 | 0.016075 | 3 | Yes |
| Cerebral midline shift | 1.3535 | 0.018243 | 4 | No |
| PT-INR | -2.1878 | 0.0428 | 5 | No |
| Hematoma density | 1.1152 | 0.0496 | 6 | No |

Note: Variables are ranked according to ascending P values. The three highest-ranked variables were retained in the parsimonious model.

**Table S2. Final three-predictor parsimonious logistic regression model.**

| **Predictor** | **β** | **SE** | **Z** | **P value** | **OR** | **95% CI** |
| --- | --- | --- | --- | --- | --- | --- |
| Smoking history (Yes) | 2.6675 | 0.5347 | 4.9884 | 0.000001 | 14.404 | 5.050–41.084 |
| Laterality (unilateral) | -2.5879 | 0.6223 | -4.1584 | 0.000032 | 0.075 | 0.022–0.255 |
| Greatest hematoma thickness | 0.1354 | 0.0377 | 3.5944 | 0.000325 | 1.145 | 1.064–1.233 |

**Model intercept:** -3.6483.

**Model equation:** logit(p) = -3.6483 + 2.6675 × Smoking history - 2.5879 × Unilateral laterality + 0.1354 × Greatest hematoma thickness.

**Coding:** Smoking history: Yes=1, No=0; laterality: unilateral=1, bilateral=0; greatest hematoma thickness was modeled as a continuous variable in millimeters.

**Table S3. Training-cohort apparent performance, discrimination, and clinical utility of the parsimonious model.**

| **Measure** | **Value** |
| --- | --- |
| Training cohort | 198 |
| Recurrence events | 33 |
| Predictors in parsimonious model | 3 |
| Events per variable (EPV) | 11 |
| AUC | 0.8955 |
| 95% CI for AUC | 0.834–0.957 |
| Optimal cutoff | 0.292 |
| Sensitivity at optimal cutoff | 0.727 |
| Specificity at optimal cutoff | 0.939 |
| Observed DCA net-benefit threshold range | approximately 0.04–0.88 |

The ROC curve, calibration curve, and decision curve analysis of the parsimonious model are presented in Supplementary Figures S1–S3.

Interpretation. The parsimonious three-predictor analysis increased the EPV to 11 while maintaining comparable apparent training-cohort discrimination. Because the ROC and DCA results above were obtained within the same training cohort used to derive the parsimonious model, they are presented as apparent performance and as a supplementary robustness assessment rather than as independent validation. The analysis does not replace the primary six-predictor nomogram or its held-out validation-cohort evaluation.

# Figures

**Figure S1. Calibration curve of the parsimonious three-predictor model in the training cohort.**

**Figure S2. Decision curve analysis of the parsimonious three-predictor model in the training cohort.**

**Figure S3. Receiver operating characteristic (ROC) curve of the parsimonious three-predictor model in the training cohort.**

# Additional sensitivity analysis using Firth penalized logistic regression

To evaluate the robustness of coefficient estimates under the limited number of recurrence events, an additional sensitivity analysis was performed using Firth penalized logistic regression. The same six predictors included in the primary nomogram were evaluated using penalized likelihood estimation. Odds ratios (ORs), 95% confidence intervals (CIs), and P values were calculated to assess whether the direction and statistical significance of the predictor associations remained consistent with the conventional logistic regression model. This analysis was performed as a sensitivity analysis and was not intended to replace the primary nomogram model.

In the Firth penalized logistic regression sensitivity analysis, all six predictors included in the primary nomogram retained consistent directions of association and remained statistically significant (Supplementary Table S4). These findings suggest that the identified predictor effects were robust despite the limited number of recurrence events.

**Table S4. Sensitivity analysis using Firth penalized logistic regression for the six predictors included in the primary nomogram.**

| Predictor | OR | 95% CI | P value |
| --- | --- | --- | --- |
| Smoking history | 7.531 | 2.853–21.270 | <0.001 |
| Unilateral laterality | 0.250 | 0.068–0.913 | 0.036 |
| Mixed hematoma density | 2.973 | 1.029–8.600 | 0.044 |
| Greatest hematoma thickness | 1.093 | 1.019–1.176 | 0.013 |
| Cerebral midline shift ≥10 mm | 3.372 | 1.187–9.804 | 0.023 |
| Postoperative pneumocephalus | 5.279 | 1.880–15.424 | 0.002 |
